# The new chronic pain MG30 category and diagnostic specificity in quality registries—problems and suggested solutions with special reference to Swedish Quality Registry for Pain rehabilitation (SQRP)

**DOI:** 10.3389/fpain.2024.1396429

**Published:** 2024-07-04

**Authors:** Emmanuel Bäckryd, Mehmed Novo, Johanna Hallsén, Stefan Schultze, Marcelo Rivano Fischer, Björn Gerdle

**Affiliations:** ^1^ Pain and Rehabilitation Center, and Department of Health, Medicine and Caring Sciences, Linköping University, Linköping, Sweden; ^2^ Department of Community Medicine and Rehabilitation, Rehabilitation Medicine, Umeå University, Umeå, Sweden; ^3^ Bragée Clinics, Stockholm, Sweden; ^4^ Pain and Rehabilitation Västmanland, Västerås, Sweden; ^5^ Department of Neurosurgery and Pain Rehabilitation, Skåne University Hospital, Lund, Sweden; ^6^ Department of Health Sciences, Faculty of Medicine, Lund University, Lund, Sweden

**Keywords:** chronic pain, code, combination, diagnosis, ICD-11, registry

## Abstract

The Swedish Quality Registry for Pain rehabilitation (SQRP) is a well-established clinical registry for adult patients with complex chronic pain conditions. SQRP registers patient-reported outcome measures from a majority of specialist chronic pain units/departments in Sweden. Up to four International Classification of Diseases version 10 (ICD-10) diagnoses can be registered in SQRP. The aim of the paper is to describe how we envision the new chronic pain category MG30 in ICD-11 can be used in SQRP. We envision that the first diagnosis in SQRP shall always be a MG30 diagnosis, which will ensure broad implementation of ICD-11 in Swedish pain care. However, at first glance, there seems to be specificity problems with ICD-11 codes that might impair their useability in SQRP or other registries. But ICD-11 offers more than meets the eye. First, the entries at the level of the so-called *foundational layer* have unique resource identifiers (URI) that can be used to enhance specificity. Second, ICD-11 contains numerous *extension codes* that can be combined with the MG30 codes – for instance, concerning the anatomical location of pain. Third, to enrich the description of the clinical concept at hand, it is possible to create *clusters of stem codes*. These three options are briefly discussed. We conclude that the full potential of the MG30 category can be better exploited in registries such as SQRP if foundational codes, extension codes, and/or clustering of stem codes are used to enhance diagnostic specificity.

## 1 Introduction

The taxonomy and classification of diseases is an area that is arguably as old as medicine itself. During the 17th and 18th centuries, emerging disease classifications were mainly symptom based (1, 2). Well-known examples are the classifications of Sauvage, Linnaeus, and Cullen. In *Genera Morborum* from 1763, Swedish botanist and physician Carl Linnaeus classified diseases in 11 categories, one of them being the painful diseases – *Morbi Temperati Nervini Sensationis Dolorosi*. The consequence of categorizing diseases based on symptoms was of course that diseases with different etiologies and pathophysiologies were often incorrectly lumped together. When French statistician Jacques Bertillon presented the precursor of what is now the International Classification of Diseases (ICD), he instead “adopted for main headings the anatomical site rather than the nature of disease” (2) thus overlooking or disqualifying pain as disease category. As of today, the structure of ICD remains overall strongly influenced by this anatomical and topographical structure. However, it is interesting to note that the eleventh version (ICD-11), adopted by the World Health Assembly in 2019, for the first time contains a structured section about chronic pain – thereby somehow reconnecting with Linnaeus' idea of pain as a separate diagnostic category.

The need for a pain classification system has long been recognized (3), and it has been argued since decades that defective and inconsistent pain taxonomies hamper the development of pain research (4). In an ideal classification system, as for instance the periodic table in chemistry, the different categories are mutually exclusive and exhaustive (5). Although this ideal will barely ever be achievable in pain medicine, the International Association for the Study of Pain (IASP) previously issued an extensive, expert-based multidimensional classification system with five axes: region of the body; organ system involved; temporal characteristics; intensity and time since onset; etiology (5). This resulted in a five-digit code, reflecting the complexity and heterogeneity of pain conditions. As of today, in countries such as Sweden where ICD-11 has not yet been implemented (it is presently being translated), diagnoses are still made according to ICD-10. Pain-relevant diagnoses are scattered throughout the ICD-10 manual and are often based on either anatomical location, duration, and/or etiology. The need for a more mechanism-based classification system has long been recognized (6, 7). A first step in that direction is the categorization of pain as nociceptive, neuropathic or nociplastic as part of an in-depth clinical assessment. This trichotomy is widely accepted by clinicians and has treatment implications, but whereas neuropathic pain is part of the ICD-11 classification scheme, the relatively new concept of nociplastic pain (8) is not.

Quality pain registries aiming to include information about diagnoses struggle with the fact that pain diagnoses are sometimes based on location, or on duration, or etiology. The aim of the present Methods paper is to describe how we envision that the new chronic pain category MG30 in ICD-11 can be used in the Swedish Quality Registry for Pain rehabilitation (SQRP).

## 2 Materials

The Swedish Quality Registry for Pain rehabilitation (SQRP) is a well-established clinical registry for adult patients with complex chronic pain conditions. SQRP registers patient-reported outcome measures (PROMs) data from a majority of specialist chronic pain units/departments in Sweden, the patients being mainly referred by primary care physicians. There are no strict inclusion criteria other than assessing that pain is chronic, with significant consequences, motivating a biopsychosocial assessment and if relevant an interdisciplinary intervention. Patients enrolled in SQRP can be characterized as complex as their health profiles often include psychiatric comorbidities such as depression and anxiety, dysfunctional coping behaviors as well as decreased working life and prolonged sick leave, low participation in social activities, and/or unresponsiveness to routine pharmacological or physiotherapeutic treatments delivered in a monodisciplinary fashion. General exclusion criteria are drug or alcohol abuse, severe psychiatric disease, pain due to a non-treated or under-treated cancer, medical conditions that do not allow physical exercise, and red flag pain conditions (i.e., other treatments are available). PROMs are completed by the patients on up to three occasions: before the first visit (baseline assessment) and for those who later participate in an interdisciplinary pain rehabilitation program (IPRP), immediately after completion of IPRP, and, finally, on follow-up one year after completion of IPRP. IPRP (also labelled as multimodal rehabilitation, multidisciplinary rehabilitation, biopsychosocial pain rehabilitation, pain management program) is an interdisciplinary intervention (with physical, occupational, psychological, social, and educational components) according to the International Association for the Study of Pain (IASP). This complex intervention is provided by a multidisciplinary team collaborating in assessment and treatment using a shared biopsychosocial approach and goals. For a detailed description of SQRP including research, see Gerdle et al. (9). There is also a primary care component in SQRP which only partly uses the same variables.

The PROMs capture a patient's background, pain intensity, pain-related cognitions, and psychological distress symptoms as well as activity/participation aspects and health-related quality of life variables. In Table 1 an overview of the variables in SQRP is presented. Diagnoses according to ICD-10 are registered in SQRP. It is possible to register up to four diagnoses in SQRP, and the first diagnosis must be a pain-related one. The most frequent ICD-10 diagnoses (first diagnosis) at baseline in SQRP are presented in Table 2. They constitute 84% of all first diagnoses registered.

**Table 1:** An overview of the mandatory SQRP variables [adapted from Gerdle et al. (9)].

Type	Variables and instruments
Self-report & background information	** **
	Sociodemographic data
	Work
	Sick leave
	Pain duration Pain extent
	Attitude towards the future
Self-report, instruments, & variables	** **
	Numeric Rating Pain Scale (NRPS)
	The Hospital Anxiety and Depression Scale (HAD)
	Multidimensional Pain Inventory (MPI)
	Health-related life quality (RAND-36)
	Perceived health (the EuroQol Group) (EQ-5D)
	Chronic Pain Acceptance Questionnaire (CPAQ 8)
	Insomnia Severity Index (ISI)
	Perceived work ability index (WAI)
	Kinesiophobia (TAMPA)
	Perceived physical activity (3 items)
	Changes in pain experience (retrospective items)
	Changes in ability to handle life situation (retrospective items)
	Patient satisfaction (6 items)
Professional-evaluated variables	** **
	Diagnosis
	Pain mechanisms
	Expected future financial-support form
	Swedish language ability
	Rehabilitation plan

**Table 2:** Most frequent ICD-10 diagnoses at assessment in SQRP.

ICD10 diagnoses	Assessment (*n* = 10,325)	Per cent (%)
M79.7	Fibromyalgia	16.9
R52.9	Pain, unspecified (generalized pain)	15.1
M79.1	Myalgia	8.3
M54.5	Low back pain	7.2
R52.2	Other chronic pain	6.7
M53.1	Cervicobrachial syndrome	4.9
M54.2	Cervicalgia	4.2
M54.4	Lumbago with sciatica	3.8
M53.0	Cervicocranial syndrome	2.6
R52.2C	Chronic pain, idiopathic^a^	2.4
R52.2A	Chronic pain, nociceptive^a^	2
M54.6	Pain in thoracic spine	1.5
Q79.6	Ehlers-Danlos syndrome	1.5
M54.9	Dorsalgia, unspecified	1.3
G44.2	Tension-type headache	1.2
M79.6	Pain in limb	1
T91.8	Sequelae of other specified injuries of neck and trunk	0.9
M35.7	Hypermobility syndrome	0.6
M51.2	Other specified intervertebral disc displacement	0.6
S13.4B	Sprain and strain of cervical spine	0.5
F43.8A	Other reactions to severe stress, Burnout syndrome	0.5
R10.2	Pelvic and perineal pain	0.5

^a^ In the Swedish version of ICD-10, R52.2 is subdivided in nociceptive (R52.2A), neuropathic (R52.2B), and idiopathic (R52.2C).

## 3 Methods

The feasibility to register not just one but several diagnoses in SQRP makes it possible to combine the new MG30 category with other ICD-11 diagnoses. In SQRP, we envision the *first diagnosis* to be a MG30 diagnosis. Making a MG30 diagnosis mandatory as first diagnosis in the registry would ensure a broad and thorough implementation of ICD-11 in Swedish pain care, starting with the units that register in SQRP. At first glance, however, there seems to be drawbacks with the MG30 category. For example, both chronic low back pain and chronic cervical pain would be coded as MG30.02 – chronic primary musculoskeletal pain. Although this may (presumably) make a lot of sense from the point of view of pathophysiology, the heterogeneity of MG30.02 (in this case concerning pain location) would nonetheless create problems registry-wise when it comes to identifying a relatively homogeneous study population. For instance, to study patients with chronic low back pain, it would not be possible to easily identify them by just selecting the ICD-11 diagnosis. Hence, there is a need for more specificity.

*Prima facie*, one solution to this problem would be to combine MG30 diagnoses with other parts of the ICD-11 system. Despite the new MG30 chronic pain category, ICD-11 retains traditional pain diagnoses outside the MG30 group, such as “low back pain, unspecified” (ME84.2Z) or “cervical spine pain” (ME84.0). Hence, such traditional diagnoses could (if used judiciously) be used as a complement in quality registries to augment diagnostic specificity. By combining a first mandatory MG30 diagnosis with a more traditional pain diagnosis as second diagnosis, “diagnostic pairs” would thereby be created. For instance, the pair MG30.02+ME84.2Z would be readily distinguishable from MG30.02+ME84.0, i.e., chronic primary musculoskeletal pain (low back pain) would be searchable and distinguished from chronic primary musculoskeletal pain (cervical back pain). Is this the way forward, or are there are alternatives available in ICD-11 to enhance diagnostic specificity in registries such as SQRP?

## 4 Anticipated results

We believe that the new MG30 category of ICD-11 has several advantages. It has the potential to increase the visibility of pain medicine as a medical specialty and academic discipline, it can help validate the experience and suffering of chronic pain patients, and it might lead to better research, e.g., when it comes to the use of registries such as SQRP. Concerning the diagnostic specificity issue described above and its *prima facie* solution, it is important to realize that ICD-11 offers more than meets the eye. By using the full potential of ICD-11, registries such as SQRP can indeed achieve higher diagnostic specificity in three ways. First, the entries at the level of the so-called *foundational layer* have unique resource identifiers (URI) that can be used to enhance specificity. Second, ICD-11 contains numerous *extension codes* that can be combined with the MG30 codes – for instance, concerning the anatomical location of pain. Third, a variant of the intuitive solution of “diagnostic pairs” already exists in ICD-11, namely the possibility to create *clusters of stem codes*. These three options will now be briefly delineated.

### 4.1 Foundational codes

A metaphor of a « shoreline » can be used to understand the importance of the foundational layer. If you are sitting in a boat looking at the coast, the visible landmass corresponds to the coding potential of the ICD-11 browser available at https://icd.who.int/en. This is the official ICD-11 for mortality and morbidity statistics (ICD-11-MMS) endorsed by the World Health Assembly (sometimes also called the “blue version” of ICD-11) (10, 11). However, there is more landmass under the surface of the water. Likewise, ICD-11 also has a “deeper” structure, called the *foundational layer,* or *the Foundations*. As expressed by Chute and Celik (12): “The Foundations functions as a deep sea of terms and meanings, where only a subset of the most common or important terms can appear on the metaphorical landmass of the linearization. The more specific terms […] are said to be “below the shoreline” of that linearization, in the depths of the Foundation.”

Importantly, every entity in the Foundations has a unique URI number. For instance, although the two primary pain conditions known as chronic low back pain and chronic cervical pain have the same ICD-11-MMS code (MG30.02), they can be differentiated by their URI: 1291385632 and 2014134682, respectively. URI:s are available at https://icd.who.int/dev11/f/en which is the so-called ICD-11 Maintenance Platform (sometimes also known as the “orange version”) (10, 11). Moreover, although fibromyalgia MG30-wise is included in the broader category of chronic widespread pain (MG30.01), fibromyalgia syndrome is still there “under the surface” and is identifiable by a more specific URI than the broader category of chronic widespread pain (236601102 and 849253504, respectively). Hence, SQRP and other registries could choose to register not only the MG30 code according to ICD-11-MMS but also the underlying URI available in the “orange version” – thereby enhancing diagnostic specificity in the registry.

### 4.2 Extension codes

A second possibility to enhance the diagnostic specificity in SQRP, e.g., to be able to differentiate between chronic low back pain and chronic cervical pain (both having the ICD-11-MMS code MG30.02), would be to use extension codes in chapter X of ICD-11-MMS. As aptly summarized by Korwisi et al. (10), “optional extension codes are available for all types of pain to document chronic pain intensity, pain-related distress, pain-related interference, the temporal course of the pain, and the presence of psychosocial factors associated with the pain”. However, from the point of view of SQRP and concerning the specificity problem which underlies the present paper, the list fails to mention one important type of extension codes, namely the possibility to use extension codes to denote anatomical location. The main categories in the extension code part of ICD-11-MMS (chapter X) are as follows (13), the asterisks indicating extension codes that we think are potentially relevant for SQRP:

• Severity scale value*
• Temporality*
• Aetiology
• Topology Scale Value
• Anatomy and topography*
• Histopathology
• Dimensions of injury
• Dimensions of external causes
• Consciousness
• Substances* (includes medicines)
• Diagnosis code descriptors
• Capacity or context
• Health devices, equipment, and supplies

Focusing on anatomical location, and using “&” as connector (14), chronic low back pain could be coded as MG30.02&XA9ET2 (i.e., with extension code for “lower back” after “&”) and chronic cervical pain as MG30.02&XA7AA6 (i.e., with extension code for “neck” after “&”). For chronic cervical pain, an alternative extension code could be XA1M78, “nape of neck”. All in all, extension codes could prove to be powerful tools to enhance diagnostic specificity in SQRP, not only concerning the anatomical location of pain but also concerning other aspects important to report, as per the list above, including the possibility to register use of opioids (under the Substances heading).

### 4.3 Clusters of stem codes

A third possibility to enhance diagnostic specificity in ICD-11 is to cluster stem codes. Any ICD-11-MMS category that can be coded on its own (a “stem code”) can also be clustered with one or more other stem codes (11). A stem code is an ordinary ICD-11-MMS code, i.e., a stem code is different from an extension code. By combining two or more codes into a cluster, the clinical concept can be more richly described (14). For chronic secondary pain conditions, normally two ICD-11 stem codes are expected: one for the chronic pain and one for the underlying disease (etiology) (10).

As of today, it is possible to register up to four diagnoses in SQRP, and the first diagnosis must be a pain-related one. Hence, while we envision that the first diagnosis in SQRP shall always be a MG30 code, the possibility remains to register other diagnoses to enhance specificity, e.g., by stating the etiology as second diagnosis. For instance, concerning chronic peripheral neuropathic pain (MG30.51), it would seem sensible to use the second diagnosis to specify etiology, e.g., postherpetic neuralgia (MG30.51/1E91.5), radiculopathy (MG30.51/8B93), or diabetic polyneuropathy (MG30.51/8C03.0), the connector “/” signifying that this is a cluster of stem codes and not an extension code (which uses “&” as connector) (14).

A final note on terminology: ICD-11 uses the term “postcoordination” both for clusters of stem codes (using the connector “/”) and for the use of extension codes (using the connector “&”) (14). Extension codes can never be used without a stem code.

## 5 Conclusion

Granted the hierarchical structure imbedded in evidence-based medicine thinking, registry cohort studies such as the ones based on SQRP cannot provide the same level of evidence as randomized controlled trials (RCT) and systematic reviews (SR). However, we argue that register cohort studies are a necessary complement to ensure that the evidence reported in RCTs and SRs also holds for a consecutive non-selected flow of patients in real-world practice settings (15). In other words, registry cohort studies can help ascertain the external validity of RCTs and SRs (their generalizability). A crucial step for registries being able to do that is the accuracy of reported diagnoses. The diagnoses listed in **Table 2** illustrate the inherent vagueness of ICD-10 concerning chronic pain, and the new MG30 categorization in ICD-11 is therefore an important clarifying step, even though only a preliminary one. As we have shown, the full potential of the MG30 category can be better exploited in registries if foundational codes, extension codes and/or clustering of stem codes are used to enhance diagnostic specificity. Moreover, from a precision medicine point of view (16), more specific diagnostic data could be used in conjunction with biomarker data to better understand the pathophysiology of different chronic pain conditions. By analyzing the multi-omic pattern of chronic pain patients with unsupervised cluster analysis (i.e., regardless of diagnosis) (17, 18), and by then comparing the frequency of diagnoses in the different clusters, it might also conceptually be possible to confirm the biological validity of traditional diagnoses and perhaps also define chronic pain conditions that are not captured well by traditional diagnoses – hence leading to future diagnostic categories that are difficult to envisage today. For instance, it is conceptually possible that what we call fibromyalgia might be a group of different diseases with only partly overlapping pathophysiology. Only the future will tell if this is so. For the time being, using ICD-11 foundational and/or extension codes and perhaps using the clustering possibility of stem codes, and combining this with the three well-known mechanistic descriptors defined by IASP (nociceptive, neuropathic, nociplastic), seems to be the most specific way to succinctly label the most common chronic pain conditions in pain quality registries such as SQRP.

## Data Availability

The original contributions presented in the study are included in the article/Supplementary Material, further inquiries can be directed to the corresponding author.
